# Supplementary material for: Aging amplifies a gut microbiota immunogenic signature linked to heightened inflammation
Source: Aging Cell. 2024 May 9;23(8):e14190. doi: 10.1111/acel.14190 (PMC11320341; doi:10.1111/acel.14190)
Supplement: Supplementary file 1 — Table S1. [file ACEL-23-e14190-s001.pdf]

Caetano-Silva et al. – Supplementary material

Aging amplifies a gut microbiota immunogenic signature linked to heightened inflammation

Table S1. Primers (Forward and Reverse, Integrated DNA Technologies, Coralville, IA) used for Fluidigm analysis.

Gene	Forward sequence	Reverse sequence
Apoe	GACCCAGCAAATACGCCTG	CATGTCTTCCACTATTGGCTCG
B3galt5	AGGCTAGTTTACGCCTCCATT	AGGAACTTCCCGTGACTTTTCT
Bcl6	CCGGCACGCTAGTGATGTT	TGTCTTATGGGCTCTAAACTGCT
Ccnd1	GCGTACCCTGACACCAATCTC	ACTTGAAGTAAGATACGGAGGGC
Ccr2	GCTGTGTTTGCCTCTCTACCAG	CAAGTAGAGGCAGGATCAGGCT
Cd55	ACCCCGGTGCATAGAGAAATC	GGATGACGTACTGTTGTCTTGG
Clca4b	TGTCACCTGGAGCAAACAAGCC	CCAGGTTGTAAGTCCAAACGCC
Cldn1	GCCATCTACGAGGGACTGTG	ACTAATGTCGCCAGACCTGAAA
Cldn2	TCTCTGTGGTGGGCATGAGA	CCAGCGGCGAGTAGAAGTC
Cldn3	ACTGCGTACAAGACGAGACG	CGGGCACCAACGGGTTATAG
Creb3l3	CAGTCAGCTCAAGAAAGCAGG	TGGTTCTGGGCAGTACACG
Cxcl10	GTGGGACTCAAGGGATCCCTC	CAGGATAGGCTCGCAGGGATG
Cxcl2	CGCTGTCAATGCCTGAAGAC	ACACTCAAGCTCTGGATGTTCTTG
Degs2	AGCGACTTCGAGTGGGTCTA	GAGAGTCCCCGTACTAACCAG
Duox2	ACCCTGGACCTCTATTCAG	ACAGCCCATTCCTAGTGT
Duoxa2	ACCGCTGCTCATTGTTATCC	AGTGCACAGCCACAATTTCG
F2	TTCGACCCCGAGGTGAAACT	CCCCGCAACATAGCACCAT
Fut1	TACCTCATCCATTGCAGACATCT	CTCCTGGGGTGATTGTCCAAG
Fut2	ACCTCCAGCAACGAATAGTGA	GCCGATGGAATTGATCGTGAA
Gda	AGGAGTGGTGCTTCAAACCAT	GGGATGAGTCAGTGTGAATTGT
Gpx2	ATGGCTTACATTGCCAAGTCG	TGCCTCTGAACGTATTGAAGTC
Hc	GAACAAACCTACGTCATTTCAGC	GTCAACAGTGCCGCGTTTT
Ido1	GCTTTGCTCTACCACATCCAC	AGCTGCCCGTTCTCAATCAG
Il10	GCTGAAGACCCTCAGGATGCG	CCTGCTCCACTGCCTTGCTCT
Il17a	ACTACCTCAACCGTTCCA	GAGGGATATCTATCAGGGTC
Il1b	GCACTACAGGCTCCGAGATGAAC	TTGTCGTTGCTTGGTTCTCCTTGT
Il22	CCTGACCAAACTCAGCAATC	GCCTTCTGACATTCTTCTGG
Il23a	CAAGGACAACAGCCAGTTCTGCTT	AGGCTCCCCTTTGAAGATGTCAGA
Il6	GACTGATGCTGGTGACAAC	ATCCTCTGTGAAGTCTCCTC
Irs1	CTCCTGCTAACATCCACCTTG	AGCTCGCTAACTGAGATAGTCAT
Itgb6	ATGGGGATTGAGCTGGTCTG	GACAGGTGGGTGAAATTCTCC
Jak3	CCATCACGTTAGACTTTGCCA	GGCGGAGAATATAGGTGCCTG
Lbp	TCCATCGGTGTCCGAGGCAAAT	AGGTCCACTGAAATGGTGACACC
Ly6a	AGGAGGCAGCAGTTATTGTGG	CGTTGACCTTAGTACCCAGGA
Ly6c1	GCAGTGCTACGAGTGCTATGG	ACTGACGGGTCTTTAGTTTCCTT

Muc1	GGCATTCGGGCTCCTTTCTT	TGGAGTGGTAGTCGATGCTAAG
Muc13	GACAGCAGTAAATGCCAGGAC	GCTGAGAGCACGTTACAGCAA
Muc2	AGGGCTCGGAACTCCAGAAA	CCAGGGAATCGGTAGACATCG
Myd88	AGGACAAACGCCGGAACTTTT	GCCGATAGTCTGTCTGTTCTAGT
Nlrp3	ATTACCCGCCCGAGAAAGG	TCGCAGCAAAGATCCACACAG
Nos2	AGTGAAAAGTCGAGCCGCA	ACAATCCACAACTCGCTCCA
Ocln	TTGAAAGTCCACCTCCTTACAGA	CCGGATAAAAAGAGTACGCTGG
Ptk6	CTCAGGCCGTGCGACATTA	GTCTTATGGTAGTCCACAAGCTC
RelA	TCCTGTTCGAGTCTCCATGCAG	GGTCTCATAGGTCCTTTTGCGC
S100a8	AAATCACCATGCCCTCTACAAG	CCCACTTTTATCACCATCGCAA
S100a9	ATACTCTAGGAAGGAAGGACACC	TCCATGATGTCATTTATGAGGGC
Saa1	GGAGTCTGGGCTGCTGAGAAAA	TGTCTGTTGGCTTCCTGGTCAG
Saa2	TGGCTGGAAAGATGGAGACAA	AAAGCTCTCTCTTGCATCACTG
Saa3	AACTATGATGCTGCCCGGAG	GCTCCATGTCCCGTGAACTT
Sbno2	TTCGCTGCGCTCAACAAGGA	TGACAGGGAATCCACAGATGAA
Sirt1	TGATTGGCACCGATCCTCG	CCACAGCGTCATATCATCCAG
Socs3	ATGGTCACCCACAGCAAGTTT	TCCAGTAGAATCCGCTCTCCT
Sphk1	ATGGAACCAGTAGAATGCCCT	TCCGTTCGGTGAGTATCAGTTTA
St8sia1	GCTACCCGTAGGAGCCAGT	CAGCACCCCTTGCACAATCT
Stat3	GTAGTGCTGCCCCGTACCTG	AGCGACTCAAACTGCCCTCC
Tff3	TCCAAGCCAATGTATGGTGCCG	CAGGGCACATTTGGGATACTGG
Tirap	ATCTCCCAGGAAAGCCACCTCT	GGTAGGTGACATTCCTGAACTGC
Tjp1	GCTTTAGCGAACAGAAGGAGC	TTCATTTTTCCGAGACTTCACCA
Tlr2	CAACAAGATCACCTACATTGGC	GACTTCAACATCAGAACCTGGA
Tlr4	CGGGAGAATCCTGTGGACAA	CTCAGACTCGGCACTTAGCA
Tnf	AGGCACTCCCCCAAAAGATG	GTAGACAGAAGAGCGTGGTGG
Tnfrsf1b	ACACCCTACAAACCGGAACC	AGCCTTCCTGTCATAGTATTCCT
Xdh	GTTGGTTTCAGCGTCAGGAG	CCCAAGTGGCAGTTTTGAGT
